# Evaluation of the Playing Time Benefits of Foreign Players in the Big-5 European Football Leagues

**DOI:** 10.2478/hukin-2022-000072

**Published:** 2022-11-08

**Authors:** Hui Zhang, Junxian Jiang

**Affiliations:** 1 Department of Sport Science, College of Education, Zhejiang University, Hangzhou, Zhejiang, China

**Keywords:** European football league, foreign players, playing time benefits

## Abstract

To explore the benefits that foreign players bring to their clubs, this study used foreign players in the Big-5 European leagues (2013/2014-2017/2018 seasons) as samples and constructed a benefit model based on playing time and game points to evaluate the contribution of foreign players to their clubs in different leagues. The results showed the following: 1) from the 2013/2014 season to the 2017/2018 season, foreign players in the Bundesliga had the highest playing time benefits (PTBs) (0.526 ± 0.012), followed by foreign players in La Liga (0.523 ± 0.014), the Premier League (0.518 ± 0.011), Serie A (0.500 ± 0.012) and Ligue 1 (0.486 ± 0.011); 2) foreign players from South America had the highest PTBs in the Big-5 leagues, while those from Africa had the lowest PTBs. However, among the different leagues, there were no significant differences in the PTBs of foreign players from the same continent; 3) PTBs of foreign players in the forward position were lower than those of foreign players in the defender position; 4) the country that produced foreign players in the Big-5 leagues with the highest total PTBs was Brazil, followed by Argentina, Spain and France. Additionally, the top 15 countries by total PTBs qualified for either the 2014 or the 2018 World Cup Final.

## Introduction

Foreign players are an important part of teams in modern football leagues. Under the recent globalization of sports, foreign players in football leagues have become an increasingly larger group (Bale and Maguire, 1994; Çobanoǧlu et al., 2018; Magee and Sugden, 2002; Radzimińskiand Jastrzębski, 2021; Taylor, 2006). With the release of the “Bosman Ruling” in 1995, football leagues of European countries have ushered in a relatively relaxed foreign players’ policy. All Big-5 leagues have removed their restrictions on European players, with some remaining for non-EU players (Simmons, 2008). This situation has led to an increase in the number of transactions in the transfer market of the Big-5 leagues, with an increasing number of foreign players playing in the Big-5 leagues (Frick, 2009; Poli, 2010; Poli et al., 2017; Storey, 2011). At the club level, the relaxation of the quota for foreign players has been beneficial. Compared with the past, clubs can use only a limited number of quotas to select foreign players, and the majority of places are reserved for domestic players. Currently, clubs have more opportunities to select quality players from around the world with limited funds, more options and more manoeuvrability.

At present, the International Federation of Association Football (FIFA) basically adopts a points system to determine the final rankings; the higher the score is, the higher the ranking. At the same time, the promotion and demotion mechanism of the rankings also determines the league level of teams in the following season. Therefore, it is very important for teams to win points during the season (Moschini, 2008), and all teams want to maximize the number of points earned in league play (Brocas and Carrillo, 2004). With the recruitment of foreign players, how to select foreign players so that teams can earn as many points as possible during playing time has become a question of interest.

Evaluations of the benefits that players bring to their teams constitute the mainstream of competitive football game research. However, in the face of complex and changeable game behaviour, researchers need to comprehensively consider various factors and make trade-offs to effectively evaluate and improve players’ performance.

There are two mainstream ways to assess the benefits that a player brings to his team. The first method is qualitative analysis, which evaluates player’s contributions. The main method is expert scoring, in which foreign players are graded by experts. Kicker publishes players’ scores every week on a scale of 1 to 6 (Deutscher and Büschemann, 2014). In addition, a number of news media provide post-game ratings of players using this method. However, the expert evaluation method has some limitations, mainly the fact that experts cannot guarantee that they are completely objective when evaluating players. The second method is quantitative analysis, which is currently used quite frequently.

Quantitative analysis is another way of assessing the benefits that a player brings to his team. Quantitative analysis is a method for establishing a mathematical model based on statistical data to obtain various indexes of objects and to quantify them using algorithms. Data comparison is the simplest way of quantitatively evaluating players’ performance. It can be carried out by collecting basic individual data on players.

In the field of football, Reilly and Thomas (1976) used notation to conduct a definitive motion analysis of soccer, they analysed in detail the movements of English First Division players using handwritten notes and tapes. Mathematical modelling has been widely accepted because of its relative objectivity. At present, researchers mainly use a variety of players’ data indicators to create behavioural indicators, multivariate statistical models and computer models (Rein and Memmert, 2016). Kempe et al. (2014) introduced a new index, the index of offensive behaviour (IOB), which combines different variables of offensive behaviour to evaluate tactical behaviour. Tiedemann et al. (2011) combined a non-concave meta-frontier approach based on data envelopment analysis (DEA) so that the influence of a player’s position can be taken into account when evaluating the player’s benefits. Duch et al. (2010) created a “ball flow” network graph analysis method based on passing between players, collecting the accuracy of passing, shooting accuracy and players’ performance distribution in Euro 2008 to quantify and evaluate performance of teams and players. Nsolo et al. (2018) analysed and compared data on players in the Big-5 leagues, screened out the attributes and outstanding techniques of players at different positions, and used machine learning to predict and evaluate players’ performance. Pariath et al. (2018) designed a computer system that could evaluate various attributes and skills of football players, and the core of the evaluation scale depended on the position of football players and the skills they had. Brooks et al. (2016) developed the PlayeRank program, a data-driven algorithm that could provide multidimensional and positional stratification for football players’ performance evaluation.

Most methods of quantitative analysis carry out mathematical modelling on data closely related to the performance of players, assign a certain weight to the data on each attribute, substitute the specific data of players into the total score, and finally produce rankings. However, the main problem of this approach is that the weight assigned to data obtained from the database may not be applicable to different sample sizes; additionally, players’ data are affected by many factors and change dynamically. Therefore, to obtain more accurate results, it is necessary to constantly improve and build the database, which is relatively complex.

The aim of this study was to use players’ playing time and game points as the main indicators to evaluate the benefits that players bring to their teams. That is, this study takes another perspective to quantify a player’s contribution to his team, providing a new way of evaluating players and enriching the players’ evaluation system.

As the most developed leagues in the world, Big-5 leagues play an important role in leading and promoting the world football (Littlewood et al., 2011; Poli et al., 2018). By exploring the playing time benefits of foreign players, we can get an insight into the pattern of foreign players in the Big-5 leagues, and better present the situation of excellent players in the world and the situation of players’ exporting countries.

## Methods

### Sample

Foreign players from the English Premier League (Premier League), France’s Ligue 1 (Ligue 1), Germany’s Fußball-Bundesliga (Bundesliga), Italy’s Serie A (Serie A) and Spain’s La Liga (La Liga) from the 2013/2014 season to the 2017/2018 season were selected as samples (Table 1). Players with no playing records were not included in the study. Foreign players with two nationalities were considered based on their first nationality. This study was approved by the local institutional ethics committee, and we obtained permission to use content from Transfermarkt.com.

**Table 1:** Numbers of foreign players in the Big-5 leagues in the 2013/2014–2017/2018 seasons.

	Premier League		Ligue 1		Bundesliga		Serie A		La Liga	
Season	*N*	*%*	*N*	*%*	*N*	*%*	*N*	*%*	*N*	*%*
2013/2014	394	70.2	311	55.7	241	50.6	325	55.4	221	40.6
2014/2015	358	65.2	303	55.3	251	54.3	332	54.5	208	39.1
2015/2016	370	66.0	301	51.1	275	55.9	327	56.1	231	42.3
2016/2017	362	66.7	284	49.4	260	54.7	330	56.7	244	44.0
2017/2018	351	66.4	290	52.4	272	56.2	303	55.1	245	42.8
$\bar{x}$	367.0	66.9	297.8	52.8	259.8	54.3	323.4	55.6	229.8	41.8

In the 2013/2014-2017/2018 seasons, among the Big-5 leagues, the Premier League had the most foreign players, with an average of 367 foreign players per season, accounting for 66.9% of the total. Serie A had the second largest number of foreign players, with an average of 323.4 foreign players per season, accounting for 55.6% of the total. Ligue 1 had the third largest number of foreign players, with an average of 297.8 foreign players per season, accounting for 52.8% of the total.

The Bundesliga had a relatively small number of foreign players (because there are only 18 teams in this league). The average number of foreign players per season in the Bundesliga was 259.8, accounting for 54.3% of the total. Among the Big-5 leagues, La Liga had the lowest number of foreign players; the average number of foreign players per season was 229.8, accounting for 41.8% of the total.

#### Playing time benefits (PTBs)

To quantitatively evaluate the PTBs of players to their teams in a season, this paper used the PTB model. The specific model is as follows:


(1) $$\text{ PTB }=\frac{10\times \sum_{i}^{n}\left(R_{i}P_{i}\right)}{N}$$

In formula (1), *PTB* is the playing time benefit, *R* is the playing ratio, *P* is the number of team points per game, *n* is the total number of games played by the team per season, *i* is the ordinal number of the game of the season (*i*= 1, 2, 3…, *n*), and *N* is the number of rounds in a season.

The playing ratio (*R*) is the ratio of the playing time of a player (*T_playe_*_r_) to the total playing time of the whole team (90 min × 11 positions). It was calculated as shown in formula (2).


(2) $$R=\frac{T_{\text{player }}}{90\times 11}$$

This study adopted the international football game points system, in which when a team won a game, drew or lost a game, it obtained 3, 1, or 0 points, respectively. The team’s achievement (points) was related to each player who played; thus, the contribution to the team could be measured by multiplying the ratio of minutes played by the team’s points (the minutes played benefit). The team’s points per game (*P*) were calculated as shown in formula (3).


(3) $$P=\left\{\begin{array}{l} 3,\text{ win }\\ 1,\text{ draw }\\ 0,\text{ lose } \end{array}\right.$$

#### Division of regions

In this paper, foreign players were divided into four segments based on their region of origin: South America, Europe, Africa and other continents (Central and North America, Asia, and Oceania).

#### Data collection and processing

The data on the playing time and game points of all players in the Big-5 leagues in the 2013/2014-2017/2018 seasons were collected from https://www.transfermarkt.com. After testing, the original data did not show a normal distribution. Therefore, we adopted a non-parametric test (Kruskal-Wallis H-test) and made pairwise comparisons; effect sizes with non-parametric tests were calculated based on the guidelines of Tomczak and Tomczak (2014).

To ensure the reliability of the data, we also collected some data from another football data website, Whoscored.com, for the consistency of testing. Since the team scoring data on the two websites were completely the same, the data collected to test the consistency were mainly based on the playing time of each foreign player in the Big-5 leagues, including the 2013/2014 Premier League season, the 2014/2015 Ligue 1 season, the 2015/2016 Bundesliga season, the 2016/2017 Serie A season, and the 2017/2018 La Liga season. The reliability of the playing time data was assessed through an interobserver testing procedure involving data from Whoscored.com. The intraclass correlation coefficient (ICC) values for inter-reliability were 1 (*p* < 0.0001), indicating that the data on the playing time of foreign players on the two websites had a very high level of consistency.

## Results

### PTBs of foreign players in the Big-5 leagues

Table 2 shows that there were no significant differences in the PTBs of foreign players in the Big-5 leagues over the five seasons examined. From the 2013/2014 season to the 2017/2018 season, foreign players in the Bundesliga had the highest PTB value (0.526 ± 0.012), followed by foreign players in La Liga (0.523 ± 0.014), the Premier League (0.518 ± 0.011), Serie A (0.500 ± 0.012) and Ligue 1 (0.486 ± 0.011). However, the PTBs of foreign players in the Big-5 leagues were very close. Foreign players in Serie A had the largest PTB value in the Big-5 leagues in the 2013/2014 season and the 2017/2018 season; foreign players in La Liga had the most PTBs in the 2014/2015 season and the 2015/2016 season, while foreign players in the Bundesliga had the greatest PTB value in the 2016/2017 season.

**Table 2:** Comparison of the playing time benefits of foreign players in Big-5 leagues in the 2013/2014– 2017/2018 seasons.

Season	Premier League	Ligue 1	Bundesliga	Serie A	La Liga	*H*	*p*	$E_{R}^{2}$
2013/2014	0.494 ± 0.024 (*n* = 394)	0.467 ± 0.024 (*n* = 325)	0.485 ± 0.025 (*n* = 311)	0.549 ± 0.030 (*n* = 241)	0.501 ± 0.031 (*n* = 221)	4.632	0.327	0.003
2014/2015	0.517 ± 0.024 (*n* = 358)	0.504 ± 0.024 (*n* = 303)	0.538 ± 0.028 (*n* = 251)	0.464 ± 0.022 (*n* = 332)	0.587 ± 0.035 (*n* = 208)	6.586	0.159	0.004
2015/2016	0.519 ± 0.023 (*n* = 370)	0.458 ± 0.024 (*n* = 301)	0.502 ± 0.027 (*n* = 275)	0.498 ± 0.026 (*n* = 327)	0.532 ± 0.031 (*n* = 231)	3.018	0.555	0.002
2016/2017	0.534 ± 0.026 (*n* = 362)	0.487 ± 0.026 (*n* = 284)	0.542 ± 0.027 (*n* = 260)	0.522 ± 0.027 (*n* = 330)	0.510 ± 0.030 (*n* = 244)	3.078	0.545	0.002
2017/2018	0.535 ± 0.025 (*n* = 351)	0.499 ± 0.026 (*n* = 291)	0.500 ± 0.026 (*n* = 272)	0.551 ± 0.030 (*n* = 303)	0.493 ± 0.029 (*n* = 245)	1.854	0.763	0.001
$\bar{x}\pm Sd$	0.518 ± 0.011 (*n* = 1835)	0.486 ± 0.011 (*n* = 1490)	0.526 ± 0.012 (*n* = 1299)	0.500 ± 0.012 (*n* = 1617)	0.523 ± 0.014 (*n* = 1149)	8.050	0.090	0.001

### PTBs of foreign players in the Big-5 leagues from different continents

The PTBs of foreign players from different continent groups were significantly different (*H* = 100.835, *p* < 0.001, $\left.E_{R}^{2}=0.014\right),$which was mainly reflected in the fact that the PTBs of foreign players from South America and Europe $(\bar{x}=0.568,Sd=$0.012 and $\bar{x}=0.538,$*Sd* = 0.008) were significantly higher than those of foreign players from Africa and other continents $(\bar{x}=0408,$*Sd* = 0.009 and $\bar{x}=$0.429, *Sd* = 0.018).

There were significant differences in the PTBs of foreign players in different leagues from different continents (Table 3). In the Premier League, foreign players from South America and Europe had the higher PTB values (0.589 and 0.544, respectively), and they were significantly better than those of foreign players from Africa and other continents (*H* = 29.859, *p* < 0.001, $\left.E_{R}^{2}=0.016\right).$There were no significant differences in the PTBs of foreign players from Africa and from other continents.

**Table 3:** Comparison of the playing time benefits of foreign players in the Big-5 leagues from different continents in the 2013/2014–2017/2018 seasons.

	South America	Europe	Africa	Other continents	*H*	*p*	$E_{R}^{2}$
Premier League	0.589 ± 0.034^Aa^ (*n* = 222)	0.544 ± 0.014^ABa^ (*n* = 1204)	0.420 ± 0.022^BCb^ (*n* = 281)	0.371 ± 0.035^Cb^ (*n* = 128)	29.859	<0.001	0.016
Ligue 1	0.673 ± 0.035^Aa^ (*n* = 224)	0.550 ± 0.026^ABb^ (*n* = 372)	0.412 ± 0.013^Cc^ (*n* = 804)	0.421 ± 0.036^BCbc^ (*n* = 89)	45.752	<0.001	0.031
Bundesliga	0.614 ± 0.038^Aa^ (*n* = 154)	0.528 ± 0.015^Aa^ (*n* = 878)	0.461 ± 0.037^Ab^ (*n* = 125)	0.474 ± 0.032^Aa^ (*n* = 142)	8.857	0.031	0.007
Serie A	0.503 ± 0.019^Aa^ (*n* = 565)	0.530 ± 0.017^Aa^ (*n* = 804)	0.397 ± 0.028^Bb^ (*n* = 218)	0.370 ± 0.068^ABab^ (*n* = 30)	15.333	0.002	0.009
La Liga	0.572 ± 0.023^Aa^ (*n* = 461)	0.551 ± 0.024^Aa^ (*n* = 434)	0.350 ± 0.024^Bb^ (*n* = 184)	0.482 ± 0.051^ABab^ (*n* = 70)	28.498	<0.001	0.025
*H*	1.48	1.582	7.446	9.215			
*p*	0.687	0.163	0.114	0.056			
$E_{R}^{2}$	0.007	0.001	0.005	0.02			

*Note: 1) Note: In each row of Table 3, values with different uppercase letters show very significant differences between groups (p < 0.01). Values with different lowercase letters show significant differences between groups (p < 0.05). Values with the same lowercase letter show no significant differences between groups (p > 0.05) (Hao and He, 2008). The same letter scheme is used in the tables below. 2) Because of the particularity of the PTBs of foreign players from other continents in Serie A, the standard deviation of their data is relatively large and the samples are relatively few. Thus, although the mean of their data is small, there is no significant difference in the PTBs of foreign players from South America, Europe and Africa*.

In Ligue 1, foreign players from South America showed the highest PTB value, 0.673, which was significantly greater than the PTB value of foreign players from Europe, Africa and other continents. The PTB value of foreign players from Europe (0.550) was significantly higher than that of foreign players from Africa (0.412) (*H* = 45.752, *p* < 0.001, $\left.E_{R}^{2}=0.031\right).$In the Bundesliga, foreign players from Africa had the lowest PTB value, 0.461, which was significantly lower than the PTB values of foreign players from South America, Europe and other continents (*H* = 8.857, *p* < 0.031, $\left.E_{R}^{2}=0.007\right).$There were no significant differences in the PTB values of foreign players from South America, Europe and other continents. In Serie A, foreign players from Africa had a relatively smaller PTB value, 0.397, which was significantly lower than the PTB values of foreign players from South America and Europe (*H* = 15.333, *p* = 0.002, $E_{R}^{2}=$0.009). Foreign players from Europe had the greatest PTB value (0.530). In La Liga, foreign players from Africa had the lowest PTB value, 0.350, which was significantly lower than the PTB values of foreign players from South America and Europe (*H* = 28.498, *p* < 0.001, $\left.E_{R}^{2}=0.025\right).$

There were no significant differences in the PTBs of foreign players in different leagues from the same continent. With regard to foreign players from South America, the league with the highest PTB value was Ligue 1 (0.673), while the league with the lowest PTB value was Serie A (0.503). Taking into account foreign players from Europe, the league with the highest PTB value was La Liga (0.551), while the league with the lowest PTB value was the Bundesliga (0.528). With regard to foreign players from Africa, the league with the highest PTB value was the Bundesliga (0.461), while the league with the lowest PTB value was La Liga (0.350). Considering foreign players from other continents, the league with the highest PTB value was La Liga (0.482), while the league with the lowest PTB value was Serie A (0.370).

### PTBs of foreign players in the Big-5 leagues at different positions

In the Big-5 leagues, the PTBs of foreign players at different positions were significantly different, which was mainly reflected by the fact that the PTBs of foreign players at the forward position were lower than those of foreign players at the defender position (Table 4).

**Table 4:** Comparison of the playing time benefits of foreign players in the Big-5 leagues at different positions in the 2013/2014-2017/2018 seasons.

	Forward position	Midfielder position	Defender position	Goalkeeper position	*H*	*p*	$E_{R}^{2}$
Premier League	0.415 ± 0.018^Bb^ (*n* = 554)	0.529 ± 0.020^Aa^ (*n* = 551)	0.579 ± 0.020^Aa^ (*n* = 569)	0.621 ± 0.046^Aa^ (*n* = 161)	45.847	<0.001	0.025
Ligue 1	0.384 ± 0.017^Cc^ (*n* = 496)	0.476 ± 0.020^Bb^ (*n* = 416)	0.570 ± 0.020^Aa^ (*n* = 498)	0.659 ± 0.071^ABab^ (*n* = 78)	51.592	<0.001	0.035
Bundesliga	0.451 ± 0.021^Cc^ (*n* = 435)	0.506 ± 0.022^BCbc^ (*n* = 365)	0.599 ± 0.021^Aa^ (*n* = 434)	0.644 ± 0.070^ABab^ (*n* = 65)	34.065	<0.001	0.026
Serie A	0.425 ± 0.023^Bb^ (*n* = 426)	0.510 ± 0.019^Aa^ (*n* = 546)	0.530 ± 0.019^Aa^ (*n* = 550)	0.598 ± 0.062^ABab^ (*n* = 95)	26.205	<0.001	0.016
La Liga	0.466 ± 0.023^Bb^ (*n* = 403)	0.476 ± 0.025^Bb^ (*n* = 301)	0.589 ± 0.023^Aa^ (*n* = 372)	0.698 ± 0.081^ABab^ (*n* = 73)	24.742	<0.001	0.022
*H*	6.965	2.763	10.079	0.772			
*p*	0.138	0.597	0.039	0.942			
$E_{R}^{2}$	0.003	0.001	0.004	0.002			

*Note: Because of the particularity of goalkeepers, the standard deviation of their data is relatively large. Thus, although the mean of their data is large, there is no significant difference from players at other positions (for example, in Serie A and La Liga)*.

In the Premier League, the PTBs of foreign players at the forward position were the smallest and were significantly lower than those of foreign players at other positions (*H* = 45.847, *p* < 0.001, $E_{R}^{2}$= 0.025). In Ligue 1, the PTBs of foreign players at the forward position were the smallest and were significantly lower than those of foreign players at other positions; additionally, the PTBs of foreign players at the midfielder position were significantly lower than those of foreign players at the defender position (*H* = 51.592, *p* < 0.001, $E_{R}^{2}=$0.035). In the Bundesliga, the PTBs of foreign players at the forward position were significantly lower than those of foreign players at the defender and goalkeeper positions; additionally, the PTBs of foreign players at the midfielder position were significantly lower than those of foreign players at the defender position (*H* = 34.065, *p* < 0.001, $E_{R}^{2}=$0.026). In Serie A, the PTBs of foreign players at the forward position were significantly lower than those of foreign players at the midfielder and defender positions (*H* = 26.205, *p* < 0.001, $E_{R}^{2}=$0.016). In La Liga, the PTBs of foreign players at the forward and midfielder positions were significantly lower than those of foreign players at the defender position (*H* = 24.742, *p* < 0.001, $E_{R}^{2}=$0.02).

There were no significant differences in the PTBs of foreign players in different leagues at the forward, midfielder and goalkeeper positions. But the PTBs of foreign players at the defender position in Serie A were significantly smaller than those of foreign players in the other four leagues (*H* = 10.079, *p* < 0.05, $\left.E_{R}^{2}=0.004\right).$

### PTBs of export countries in the Big-5 leagues

**Figure 1:**
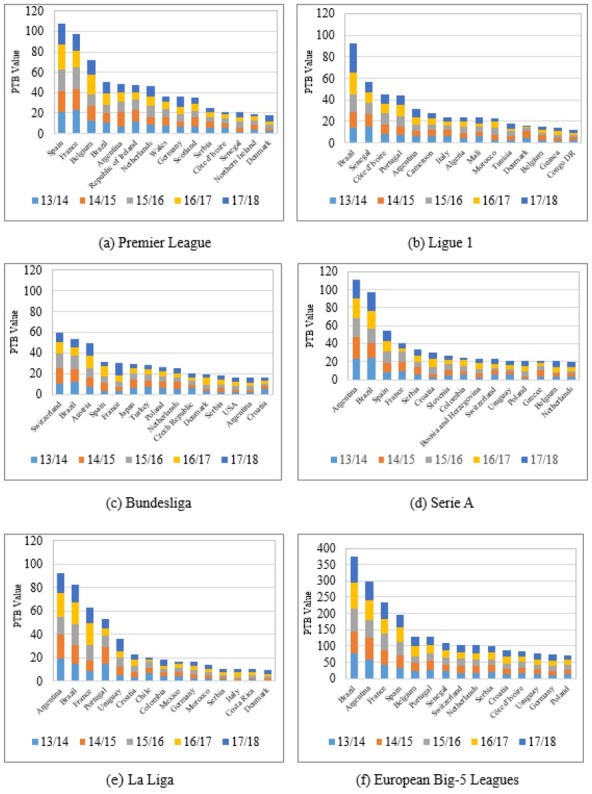
The PTBs of export countries in the Big-5 leagues during the 2013/2014–2017/2018 seasons.

This paper summarizes the PTBs of foreign players in the Big-5 leagues to explore the benefits of foreign players to these five leagues. Figure 1 illustrates the rankings of export countries by PTBs in the Big-5 leagues (top 15 countries/regions). In the Premier League, the top five countries by foreign player PTBs were Spain, France, Belgium, Brazil and Argentina (Figure 1a). In Ligue 1, the top five countries were Brazil, Senegal, Côte d'Ivoire, Portugal and Argentina (Figure 1b). In the Bundesliga, the top five countries were Switzerland, Brazil, Austria, Spain and France (Figure 1c). In Serie A, the top five countries were Argentina, Brazil, Spain, France and Serbia (Figure 1d). Finally, in La Liga, the top five countries were Argentina, Brazil, France, Portugal and Uruguay (Figure 1e).

Among the Big-5 leagues, the PTBs of foreigner players from different countries/regions varied (Figure 1f). Players from Brazil made the greatest contribution to their teams in the Big-5 leagues, with a total PTB value of 375.928, ranking among the top for each season and showing an increasing trend year by year. Players from Argentina made the second greatest contribution; their total PTB value was 298.692, which was relatively stable each season. France ranked third; the total PTB value of foreign players from France (i.e., excluding French players in Ligue 1) was 232.983, and it has shown a rising trend in recent years. Spain ranked fourth; the total PTB value of foreign players from Spain (i.e., excluding Spanish players in La Liga) was 196.717. The rest of the top 15 countries in the Big-5 leagues were Belgium, Portugal, Senegal, Switzerland, the Netherlands, Serbia, Croatia, Ivory Coast, Uruguay, Germany and Poland. Players from these countries also significantly contributed to their teams in the Big-5 leagues over the past five seasons.

## Discussion

### Playing time benefits (PTBs)

In this paper, the contribution of foreign players to their teams was measured by establishing a PTB model, which was mainly composed of playing time and game points. Although these two indexes obscure the importance of the positions of players on the field and specific technical and tactical behaviours, they are both core indexes and basic indexes in football matches_._ Thus the PTB model can better reflect the quality (points) and quantity (playing time) of players participating in matches.

For teams, the minutes played by all players in a season are constant (not counting injury time) and equal to 90 minutes multiplied by 11 position times and the total number of games played. During a season, every team needs to allocate these minutes to its players. The total amount of playing time is the same regardless of how it is allocated. A team allocates playing time to players in the hope of gaining as many points as possible. From a certain perspective, when a team gives players a certain amount of playing time, players with high PTBs have a better chance of scoring as many points as possible for the team compared to players with low PTBs. A football game is a team competition, and the processes of the game are linked together. The final result is a function of every player on the field. Just having the players on the pitch is part of the game, and everything they do can change the outcome. Therefore, the final points of a game are also closely related to every player on the field, which can be a macro evaluation of the benefits that players bring to their teams.

Playing time and game points are applicable to all players, and the evaluation criteria are consistent. Therefore, the PTB model enables players from different leagues, at different positions and of different nationalities to be compared and analysed from a macro perspective. At the same time, the PTBs of foreign players can reflect the performance of players to some extent because the performance of players and their playing time are closely related. From the perspective of resource allocation efficiency, a game can involve a maximum of only 14 players (11 starters and 3 substitutes), which means that some players of a team are not allowed to play. The most direct determinant of a player’s availability and playing time is the head coach. The coach tries to help his team earn points in every game. The coach usually selects players who are the best at their position or who can be helpful in a game. The replacement of players and allocation of playing time to players are also based on the performance of players on the field and technical and tactical needs. Therefore, a player’s performance is closely related to his playing time. The player who performs better in a game will receive more playing time and thus, more effective his playing time will be.

To verify the applicability of the player’s PTB model, this paper collected some technical and tactical indicators related to each player position in the season from Whoscored.com (goalkeepers: total clearances, total saves, aerials won, total passes, total key passes, and total assists; defenders, midfielders and forwards: total tackles, total interceptions, the number of times fouled, the number of fouls, total clearances, total blocks, total shots, total goals, successful dribbles, aerials, total passes, total key passes, and total assists). The Technique for Order Preference by Similarity to an Ideal Solution (TOPSIS) is used to obtain total scores for each player. Then, the PTBs were analysed and compared based on the positions of players, the results are shown in Table 5. In the selected samples, the PTBs of foreign players at different positions and the TOPSIS scores obtained using the players’ technical and tactical indicators were all significant at the 0.01 level, and r values were all above 0.690, indicating strong correlations. To some extent, these results demonstrate that the PTB model can be used to evaluate the contribution of players to their teams.

**Table 5:** Correlation analysis of players' playing time benefits and TOPSIS scores.

	Premier League (13/14 season)	Ligue 1 (14/15 season)	Bundesliga (15/16 season)	Serie A (16/17 season)	La Liga (17/18 season)
Goalkeepers	0.770^**^ (*n* = 32)	0.759^**^ (*n* = 14)	0.690^**^ (*n* = 18)	0.758^**^ (*n* = 20)	0.787^**^ (*n* = 18)
Defenders	0.855^**^ (*n* = 118)	0.794^**^ (*n* = 98)	0.779^**^ (*n* = 96)	0.873^**^ (*n* = 106)	0.730^**^ (*n* = 72)
Midfielders	0.883^Aa^ (*n* = 127)	0.895^**^ (*n* = 84)	0.852^**^ (*n* = 72)	0.860^**^ (*n* = 113)	0.843^**^ (*n* = 68)
Forwards	0.866^**^ (*n* = 117)	0.901^**^ (*n* = 107)	0.897^**^ (*n* = 89)	0.917^**^ (*n* = 91)	0.904^**^ (*n* = 87)

** indicates a correlation significant at the 0.01 level

### Contribution of foreign players in the Big-5 leagues from different continents

The comparison of the PTBs of foreign players in the Big-5 leagues from different continents showed that foreign players from South America made the greatest contributions, while foreign players from Africa and other continents made smaller contributions. Comparing the PTBs of foreign players from the same continent in different leagues, we found that players from South America were relatively better in Ligue 1 and the Bundesliga. Players from Europe performed better in Ligue 1 and La Liga. Players from Africa were relatively better in the Bundesliga. Players from other continents performed better in La Liga and the Bundesliga. These results can be considered when introducing foreign players from different continents.

### Contribution of foreign players in the Big-5 leagues at different positions

This study found that foreign players at the goalkeeper position and the defender position were more effective in their playing time, while foreign players at the forward position were less effective in their playing time. This finding is consistent with reality; the main reason is that modern football emphasizes offense because teams are more inclined to reserve a limited number of substitutes for players at the forward position to strengthen their offense. With regard to defenders and goalkeepers, compared with midfielders, due to the limited number of places, these players receive fewer substitute opportunities and usually play the whole game. Since they receive more substitute opportunities, foreign players at the forward position have less playing time in a season than those at the defender and goalkeeper positions, and as a result, the PTBs of foreign players at the forward position are smaller than those of foreign players at the goalkeeper and defender positions. This finding fully reflects the fact that the Big-5 leagues focus on offense, use substitutes for players at the forward position, maintain the offensive power of the forward position, and hope to score more goals in their games.

### Nationality preference for foreign players of each league

The PTBs of countries in the Big-5 leagues seem to reflect the nationality preference for foreign players of each league. Players from Brazil and Argentina are favoured in the Big-5 leagues, and this situation seems to be common in every major league worldwide. Most players in the Big-5 leagues are from the EU countries. Firstly, from the perspective of the geographical location, European countries are closely linked, making it easier to move players from one country to another. It does not take a long time for a player to travel from home to the league host. Secondly, most European countries are developed countries with similar living standards and lifestyles; thus, players can quickly adjust to local life, reducing their homesickness to some extent. Finally and most importantly, the release of the “Bosman Ruling” ensures that players from EU countries are not subject to the restrictions on foreign players in the Big-5 leagues. This situation has greatly reduced the competitive pressure on players from EU countries and has made such players very active in the European transfer market (Maguire and Stead, 1998). At the same time, it has also given league clubs more options in the transfer market, and clubs do not need to take into account the quotas for foreign players when recruiting EU players in the transfer market. It is noteworthy that of the top 20 countries from which Ligue 1 draws players, the African nations of Senegal, Côte d'Ivoire, Cameroon, Algeria, Mali, Morocco, Tunisia, Guinea, and Togo are all former French colonies.

### Relationship between a country’s PTBs and national teams’ performance

As illustrated in Figure 1, all of the countries on the list of the top 15 countries in terms of the total PTBs of foreign players in the Big-5 leagues in the 2013/2014–2017/2018 seasons have world-famous football teams. Additionally, the top 15 countries by total PTBs qualified for either the 2014 or the 2018 World Cup Final. To further explore whether there is a relationship between the national PTBs of foreign players in the Big-5 leagues and the world rankings, this paper conducted a correlation analysis between the national PTBs of foreign players in the Big-5 leagues and their national team’s world ranking points (data released by FIFA in June 2014-2018). The results were significantly correlated with each other (r = 0.654, *p* < 0.001). In other words, the higher the total PTBs of foreign players in the Big-5 leagues, the higher the scores of their national teams should be. The countries of total PTBs of foreign players in 2013/2014, 2014/2015, 2015/2016, 2016/2017, and 2017/2018 seasons were highly correlated with their world ranking points (r = 0.615, 0.647, 0.641, 0.702, and 0.661, respectively; *p* < 0.001, Table 6).

**Table 6:** Correlation analysis of the PTBs in the Big-5 leagues and world ranking points in the 2013/2014–2017/2018 seasons.

	2013/2014	2014/2015	2015/2016	2016/2017	2017/2018
World ranking points	0.615^**^	0.647^**^	0.641^**^	0.702^**^	0.661^**^

** indicates a correlation significant at the 0.01 level

However, it is unclear whether foreign players with better PTBs will boost performance of their national teams or whether foreign players from the national team at a higher level will be more effective on the field. Lago-Peñas et al. (2019) have argued that the relationship between foreign players and the ranking of the exporting national team is very complex and that the causal relationship is mainly reflected from the football performance level of the national team to the output of excellent football players to the league, which is an immediate effect. However, the improvement in the level of the national football team due to the feedback of excellent foreign players in the Big-5 leagues is a highly time-consuming process of accumulation.

## Conclusions

From the 2013/2014 to the 2017/2018 season, foreign players in the Bundesliga had the highest PTB value, followed by foreign players in La Liga, the Premier League, Serie A and Ligue 1. Foreign players from South America had the highest PTBs in the Big-5 leagues, while foreign players from Africa had the lowest PTBs; however, there were no significant differences in the PTBs of foreign players in different leagues from the same continent. The PTBs of foreign players at the forward position were lower than the PTBs of foreign players at the defender position. The country with the highest total PTBs of foreign players in the Big-5 leagues was Brazil, followed by Argentina, Spain and France. Additionally, the top 15 countries by total PTBs qualified for either the 2014 or the 2018 World Cup Final.
